# A Telemetry System Embedded in Clothes for Indoor Localization and Elderly Health Monitoring

**DOI:** 10.3390/s130911728

**Published:** 2013-09-04

**Authors:** Yoann Charlon, Nicolas Fourty, Eric Campo

**Affiliations:** 1 CNRS, LAAS, 7 Avenue du Colonel Roche, Toulouse F-31400, France; E-Mail: campo@laas.fr; 2 Univ de Toulouse, UPS, UTM, LAAS, Toulouse F-31400, France; 3 LCIS, Grenoble-INP, Université Pierre Mendès, 50, rue Barthélémy de Laffemas BP54–F-26902 Valence, France; E-Mail: nicolas.fourty@iut-valence.fr

**Keywords:** wireless sensors, telemetry, low power, ultrasonic, localization, 802.15.4, elderly health monitoring

## Abstract

This paper presents a telemetry system used in a combined trilateration method for the precise indoor localization of the elderly who need health monitoring. The system is based on the association of two wireless technologies: ultrasonic and 802.15.4. The use of the 802.15.4 RF signal gives the reference starting time of the ultrasonic emission (time difference of arrival method). A time of flight measurement of the ultrasonic pulses provides the distances between the mobile node and three anchor points. These distance measurements are then used to locate the mobile node using the trilateration method with an accuracy of a few centimetres. The originality of our work lies in embedding the mobile node in clothes. The system is embedded in clothes in two ways: on a shoe in order to form a “smart” shoe and in a hat in order to form a “smart” hat. Both accessories allow movements, gait speed and distance covered to be monitored for health applications. Experiments in a test room are presented to show the effectiveness of our system.

## 1. Introduction

The aging population context in developed countries has highlighted several economic and societal problems over last decades. Recent advances in the fields of information technology, sensor networks and miniaturized devices are offering new technological solutions for taking care of the elderly. The research and development of smart monitoring systems, the purpose of which is to care for the elderly living at home or in institutions, has been the subject of considerable effort in academia and certain industries [1,2]. Some of these systems enable monitoring of activities of daily living (ADLs) [3,4]: life habits, physical activity, gait speed, *etc.* In order to monitor ADLs, some research teams use sound or video [5,6]. These options have been criticized and described as an intrusion in terms of patients' privacy. Other research teams have used non-intrusive sensors based on motion detection. They generally use sensor networks disseminated in the environment or worn by the person (on clothes, as implants, *etc.*), infrared sensors [7–9], inertial sensors [10,11], ultrasonic sensors [12,13], or floors equipped with pressure sensors [13,14].

In our work we have chosen to use both non-intrusive sensors worn by the person and disseminated in the environment for monitoring ADLs at home or in hospital wards [15,16]. This relates to one of our previous studies [16] in which we developed a telemetry system in order to measure the distance between two devices with an accuracy of a few centimetres.

This paper is focused on the integration of our prototype into clothes. The main objective is to provide a “smart” garment for monitoring ADLs in institutions or at home. Indeed, by combining this garment with a network of anchor points, it is possible to monitor the movements of a person in his/her indoor environment and in real time. Moreover, a post-processing analysis allows measurement of gait speed and the distance covered by the user. Based on these parameters, many clinical applications are possible. The monitoring of physical activity and walking trajectories allows observing the evolution of Alzheimer's disease in medical institutions [15]. We also plan to use our instrument to follow up on frail subjects during their daily life at home. Frailty [17] is a syndrome determining a higher vulnerability to stressors and responsible for an increased risk of major negative health-related events, including disability. Numerous factors link the concept of frailty to physiological and clinical indicators (e.g., gait speed, physical activity, *etc.*). Therefore, a specific need is the development of a methodology allowing a dynamic and objective assessment of key parameters to be followed up over time. The measurement of gait speed and its variability, distance covered and daily activities may indeed provide useful indicators to generate different clinical trajectories and allow the design of specifically tailored interventions.

The system presented here is a precise telemetry system composed of four devices: a first node, which is an energy efficient mobile device (called “Telemeter”) and worn by a person on a shoe or in a hat, and three anchor points (called “Beacons”). These Beacons allow the person wearing the device to be located precisely in an indoor environment using a trilateration method. Finally, a local unit enables observation of movements in real time and saves the data in a database. These data are treated in order to compute walking trajectories, the gait speed and the distance covered by the user.

In this paper, we first present the basic principles of the proposed system, then hardware and software development are described. Finally, electrical and localization characterization results are presented.

## 2. Localization and Navigation System

### 2.1. Related Work and Technical Choices

Location tracking techniques can be classified into two categories [18]:

- active systems with devices worn by the person and embedded in the environment;
- passive systems with devices embedded in the environment.

The deployment of a localization system at home must be fast and simple. It seems difficult to use a passive localization system at home because its deployment requires a large number of nodes and a complex calibration (e.g., fingerprinting method) [18]. Active systems provide wide coverage, limited infrastructure requirements, simple calibration, and cost-effectiveness. Moreover, an active system enables identification of the user, which is essential in a multi-user environment. It is also possible to add some sensors, such as accelerometer in order to measure the ADLs with greater accurate (e.g., helpful to measure a stationary activity such as exercise bike). The main issue of active systems is the necessity to wear a device. In order to be accepted by the user, the wearable system must be miniaturized. In this work, we used an active system in order to propose simple and fast installation at home.

In active systems, two methods are predominantly used to calculate the position of a mobile node in an indoor environment: triangulation [19] and trilateration [20]. Triangulation uses the measurement of angles between the mobile node and certain anchor points. This method requires complex hardware devices such as unidirectional antennae [19], increasing the cost of the system (e.g., an indoor GPS system with four transmitters and one receiver costs up to $ 45,000 [21]). Trilateration uses the measurement of distances between the mobile node and certain anchor points. The estimation of distances can be accomplished with simple hardware such as omnidirectional antennae, which is cheaper than triangulation (e.g., an indoor localization setup based on the Cricket system with four transmitters and one receiver costs $ 1,000). This option was chosen for its low cost and also because the deployment of this type of system at a real site is easier.

In the trilateration method, three techniques tend to be used to measure the radial distance between the mobile node and each anchor point: the received strength signal index (RSSI) method [22–24], the time of arrival (ToA) method [25,26], and the time difference of arrival (TDoA) method [26,27]. The RSSI method is not suitable for our application because the localization accuracy is low (a few metres) [22–24]. It has been shown that systems using ToA and TDoA methods can achieve an accuracy of a few centimetres [26]. The common principle of these methods is the time of flight (ToF) measurement of an acoustic or electromagnetic wave to infer the distance between two devices. The ToF measurement of an electromagnetic wave that travels at high speed (3 × 10^8^ m/s) requires a high precision electronic instrument working at high frequency in order to achieve good resolution. This implies a high cost. Thus, several low-cost systems exploit the propagation of an ultrasonic wave [26,27] which travels much slower than an electromagnetic wave (approximately 1 million times).

These low cost systems exploit the propagation speed difference between the electromagnetic and ultrasonic waves (TDoA method). The electromagnetic signal (RF or light) gives the reference time in order to measure the ToF of the ultrasonic pulses. The most common indoor localization systems which use TDoA method are:

- the Active Badge system, using infrared and ultrasonic signals [26,28];
- the Bat system, using RF and ultrasonic signals [26,29];
- Cricket from MIT, using RF and ultrasonic signals [26,27].

Such low-cost and accurate localization systems are suitable for an application monitoring the elderly at home. In a first step, we have developed a telemetry system in order to measure the distance between two devices with an accuracy of a few centimetres [16]. The characterization of ultrasonic performance shows good reliability, linearity and multipath immunity. This system has also been tested and compared with the MIT Cricket system [27] and has demonstrated several advantages [16] such as:

- better accuracy (from 5% to 10% in the worst case and over 3 m);
- better opening angle (90° from the direct path);
- better maximal range (up to 10 m);
- better energy efficiency: the 802.15.4 low power modes operate for up to two weeks with standard alkaline batteries (for a measurement every second).

The performances of this telemeter allow us to envisage several applications. The goal of this paper is to establish an indoor localization system with several telemeters in order to test these performances in the context of monitoring the elderly at home.

### 2.2. Operating Principle

**Figure 1.**
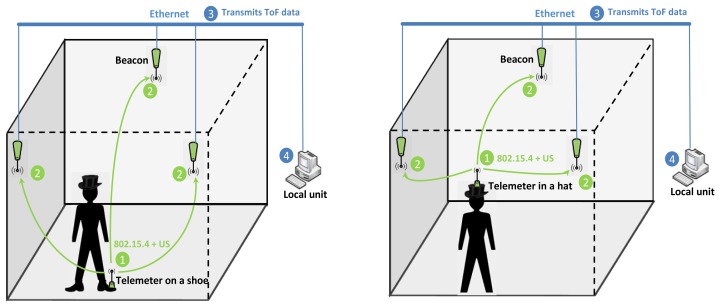
System architecture: Telemeter embedded on a shoe or in a hat.

The system has two main functionalities. The first is to estimate the position of the user, typically a pedestrian in an indoor environment, and the second is to calculate the movements of the user. The telemetry system is embedded on the person in two ways: on a shoe in order to form a “smart” shoe and in a hat in order to form a “smart” hat. The system architecture is shown in Figure 1. Data collection involves several stages:

- Stage 1: the Telemeter sends a radio message using the 802.15.4 interface [30] and an ultrasonic pulse simultaneously every 256 ms (TDoA method). The radio frame is presented in Section 4.
- Stage 2: the Beacons receive the radio message and start a timer which measures the ToF of the ultrasonic pulse. When the ultrasonic pulse reaches a Beacon, an interrupt is generated in order to compute the ToF.
- Stage 3: ToF data received by each Beacon are sent through the Ethernet network to the local unit (processing terminal). We use the Ethernet network in order to deploy our system rapidly in the laboratory room. The 802.15.4 interface of our system could be used to send the ToF data to the local unit with several advantages: the wireless system, low power consumption, the ease of creating a mesh network, and the support for large number of nodes. This option will be studied in the case that our system is deployed at a real site.
- Stage 4: a real time application installed on the local unit allows computation of the position of the Telemeter using a trilateration method. Each position is recorded and dated in a database. From these position data post-processing software calculates the movements of the user: gait speed, distance travelled and trajectories.

### 2.3. Distance Estimation

The system is based on the association of two wireless technologies: ultrasonic and 802.15.4. A ToF measurement provides the distance between two devices. The Telemeter sends a radio message simultaneously and periodically using the 802.15.4 interface and an ultrasonic pulse. The temporal management of the ultrasonic and radio signals has been detailed in a previous publication [16]. The speed of the radio signals is approximately 3 × 10^8^ m/s, whilst the speed of sound through air is approximately 3.4 × 10^2^ m/s. The propagation time of radio signals being much higher than the ultrasonic propagation time, the ToF of the RF wave can be considered as instantaneous. The use of the RF signal gives the reference time in order to measure the ToF of the ultrasonic pulses. The distance D between the two devices is computed thus:

(1) $$D=(T_{\text{Fus}}-\text{offset})\cdot V_{us}$$

where T_Fus_ is the measured ToF of the ultrasonic pulse, offset is a delay determined empirically and caused by the conditioning electronic circuit, and V_us_ is the speed of sound, which depends on the temperature of the room. The speed of sound through air is approximately 331.5 m/s at 0 °C, and 343 m/s at 20° (normal room temperature). At normal atmospheric pressure, the temperature dependence of the speed of a sound wave through air is approximated by the following equation:

(2) $$V_{us}=331.5+0.6T$$

where T is the temperature of the air in degrees Celsius. A temperature sensor has been integrated in the telemetry system in order to correct the variation of the speed of sound through air.

### 2.4. Localization Method

In a 3D space, trilateration requires four measured distances between the mobile node and the anchor points. The mobile node is at the intersection of four spheres whose geometric centres are the anchor points and their radii are the measured distances. The geometric problem of trilateration in a 3D space is articulated in a previous publication [20]. However, when the schema of the anchor points is adequate, only three sets of distance measurements are needed to find the 3D position of the mobile node [20]. Indeed, three anchor points define three spheres with two solutions. If the anchor points are attached to the ceiling, one of the solutions is located above the ceiling, while the other solution is located beneath it. The first can be discarded because ultrasonic does not go through walls.

**Figure 2.**
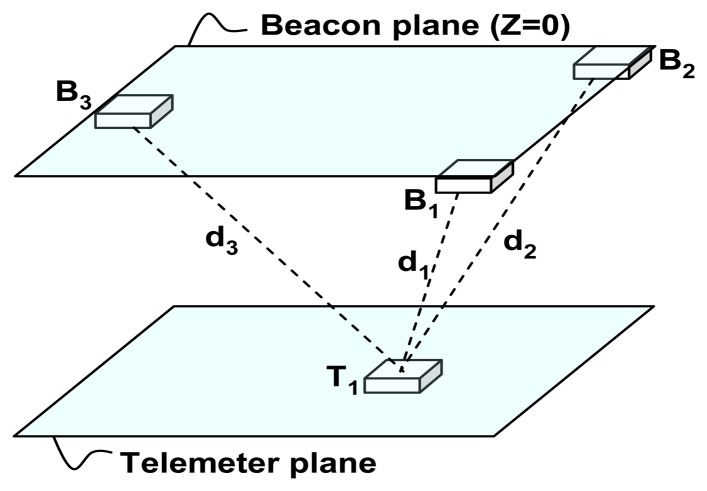
Layout of 3D localization system.

In this study, three anchor points are used to extract the position of the mobile node in a 3D indoor environment. Figure 2 shows three anchors points called Beacons (B1, B2 and B3) that act as reference points in a known coordinated system. The mobile node (Telemeter T1) transmits RF and ultrasonic signals and all the Beacons can determine their own distances relative to the Telemeter position by measuring the ToF of the US signal. The distance measurement is performed by estimating the distance between the Telemeter and the Beacons within the coverage area under the ceiling.

**Figure 3.**
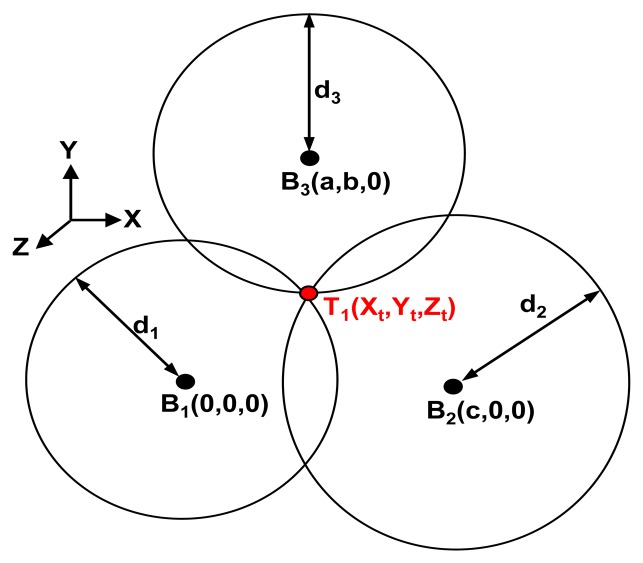
2D layout of the 3D localization system.

In Figure 3, the 2D layout of the 3D localization system is shown. Telemeter T_1_ is in a certain position (X_t_, Y_t_, Z_t_), and the Beacons are at the points B_1_ (0, 0, 0), B_2_ (c, 0, 0) and B_3_ (a, b, 0). The distances between the Telemeter and the Beacons are d_1_, d_2_ and d_3_, three spheres are defined.

The equation arrays of the three spheres are defined as:

(3) $$\begin{array}{ll} {d_{1}}^{2} & ={X_{t}}^{2}+{Y_{t}}^{2}+Z_{t}^{2}\\ {d_{2}}^{2} & =(X_{t}-c)+{Y_{t}}^{2}+{Z_{t}}^{2}\\ {d_{3}}^{2} & (X_{t}-a)+(Y_{t}-b)+{Z_{t}}^{2} \end{array}$$

This equation array can be solved by clearing the variables X_t_, Y_t_ and Z_t_. The 3D coordinates of the Telemeter are defined as:

(4) $$\begin{array}{ll} X_{t} & =\frac{{d_{1}}^{2}-{d_{2}}^{2}+c^{2}}{2c}\\ Y_{t} & \frac{{d_{1}}^{2}-{d_{3}}^{2}+{\left(X_{t}-a\right)}^{2}}{2b}+\frac{b}{2}-\frac{{\left({d_{1}}^{2}-{d_{2}}^{2}+c^{2}\right)}^{2}}{8bc^{2}}\\ Z_{t} & =\sqrt{{d_{1}}^{2}-{X_{t}}^{2}-{Y_{t}}^{2}} \end{array}$$

### 2.5. Displacement Estimation

The main objective is to measure the mobility development of frail elderly people in their living place in order to ensure continuity of medical care. Gait speed and distance covered allow quantification of mobility. These parameters will be averaged by period (day, week, and month) in order to reflect the mobility development of the people over a long-term period.

#### 2.5.1. Estimation of Speed and Walking Distance in Real Time

The localization system is clocked by the sending of a positioning request by the Telemeter every 256 ms. Each new position is dated and recorded in the database. The distance travelled D_t_ at the t time is calculated using the six past positions (x, y) recorded and is computed thus:

(5) $$D_{t}=\overset{k=5}{\underset{k=0}{\text{∑}}}\sqrt{{\left(x_{k}-x_{k-1}\right)}^{2}+{\left(y_{k}-y_{k-1}\right)}^{2}}$$

The average speed V_t_ at the t time is defined as:

(6) $$V_{t}=D_{t}*\Delta T$$

where ΔT is the time elapsed between the six past positions recorded. Real tests have shown that taking more than six past values (1.5 s) into account is not suitable due to possible actual speed variations. Indeed the gait speed variation is greatly decreased with a sliding window more than 1.5 s.

#### 2.5.2. Detection of Walking Periods

This first estimate of the speed is used to assess whether the person is in motion. A condition for moving and stopping has been established empirically. If the average speed V_t_ is greater than 0.2 m/s, movements are taken into account. When the speed drops below 0.2 m/s, the stop condition is activated and the path performed by the user is registered. This condition prevents displacements being taken into account if there are positioning errors in the static position.

#### 2.5.3. Post-Processing

When the path has been recorded, a post-processing is used to filter measurement points that are inconsistent with the displacement recorded. These errors are related to obstruction of the signal by the user and synchronization misalignment (see type 1 and 2 errors in Section 5.2.1). A first filtering is performed on the set of points by calculating the Gaussian distance deviations with a confidence interval of 95%. The measurement points that are too far from others are removed.

**Figure 4.**
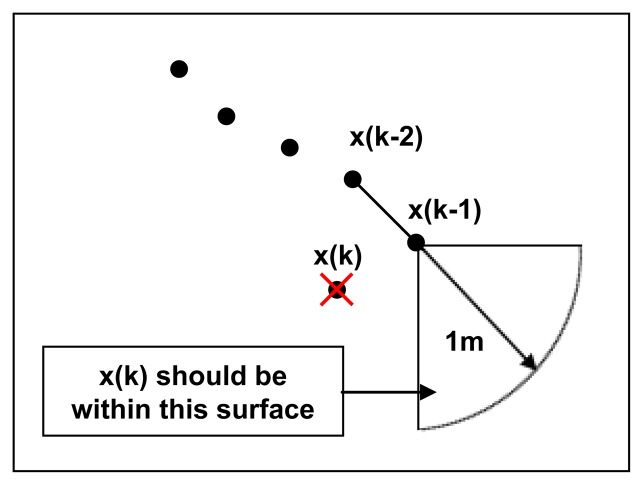
Smoothing walking paths.

A second treatment smoothing walking trajectories is illustrated in Figure 4. We use an approach based on supposing that the person is going to follow the straight trajectory defined by the line that joins the last two estimated positions [x(k−2) and x(k−1)] in the same direction. Hence, the next position x(k; can be corrected by applying some basic geometrical laws defined empirically with the first tests of our system:

1. The maximum change of direction allowed can be up to 45° relative following a straight line.
2. The maximum distance between two measurement points may not exceed 1 m.
3. When a position is not situated on the surface defined by geometrical laws (1) and (2), the position x(k) is deleted and the filtering restarts at the position x_0_.

#### 2.5.4. Estimation of Speed and Walking Distance after Post-Processing

When the trajectories were smoothed, average speed and distance covered were recalculated for the entire path. The total distance is calculated using formula 5, where the index k is equal to the total number of positions on the path. The average speed is calculated using formula 6, where ΔT is the total elapsed time of the path.

## 3. Hardware Presentation

### 3.1. Prototype Architecture

**Figure 5.**
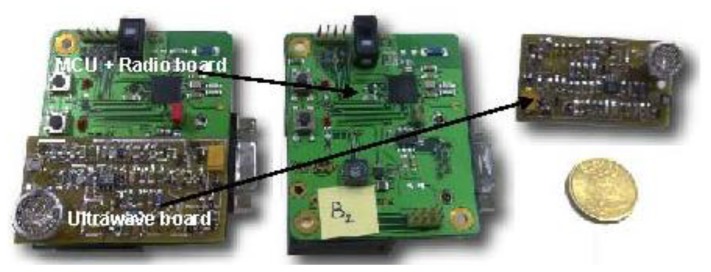
RF and ultrasonic devices.

The prototype (Figure 5) comprises two parts separated in two specific boards connected through dedicated Programmable Input/Output (PIO). The first board contains the MicroProcessor Unit (MPU) and a radio modem, while the second board is dedicated to the ultrasonic emission/reception pulses. The details of the hardware architecture and the system block diagram have previously been presented [16].

The system is based on the association of two wireless technologies: ultrasonic and RF 802.15.4. The MPU part aims to handle the 802.15.4 transceiver and command the application. The ultrasonic part aims to compute the ToF of an ultrasonic pulse in the 40 kHz frequency range.

The prototype can be configured in Beacon mode or in Telemeter mode using dedicated programs (see Section 4). In Beacon mode, the system is fixed to a wall and is powered by a 9 V battery or by the electrical grid. In Telemeter mode, the system is worn by the user and is powered by a 9 V battery.

### 3.2. Ethernet Board

**Figure 6.**
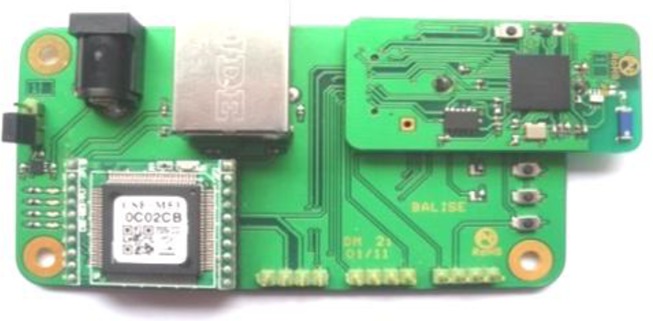
Ethernet board.

Data received by the Beacon are sent to the local unit via an Ethernet board configured in TCP/IP. This board also has an RF 802.15.4 wireless transceiver. Figure 6 shows the Ethernet board used.

### 3.3. Telemeter Embedded in Clothes

**Figure 7.**
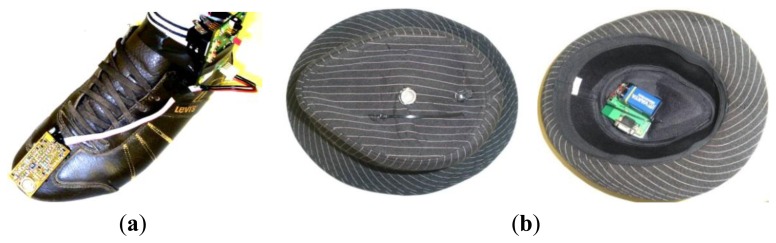
(**a**) Device embedded on a shoe. (**b**) Device embedded in a hat.

The Telemeter is embedded in clothes in two ways: on a shoe in order to form a “smart” shoe and in a hat in order to form a “smart” hat, as shown in Figure 7.

## 4. Software Presentation

In order to program the application easily, Freescale offers several software solutions called Code Bases: a basic solution called SMAC, a more complex 802.15.4 compliant stack and a ZigBee compliant stack [31].

For our system, we chose the basic Simple Media Access Controller (SMAC) for several reasons. The most important is that this code base is completely open source and gives access to very low level primitives enabling maximal energy savings. Moreover, this code base is small and easy to implement. The source code is in standard C language and the development environment is Code Warrior [32].

The application software of Telemeter and Beacon were modified on the basis of previous research [16] in order to save energy on the Telemeter application embedded in clothes. The computation of ToF that allows determination of the distance between Beacons and the Telemeter is now performed using fixed Beacons which are connected to the electrical grid. This software is optimized for a single user at home. Interference tests were conducted in previous research [16]. The conclusion of this study is that position errors can occur when more than two telemeters communicate. In a multi-user environment, a synchronization protocol combined with an adapted medium access control algorithm could lower the interference issues from several telemeters.

### 4.1. Application Software

The application code is integrated in a state machine running on the Beacon and the Telemeter. Localization requests are undertaken periodically (256 ms) using a timer on the Telemeter.

#### 4.1.1. Telemeter software

In order to save energy, the system spends most of its time in a deep sleep mode called Hibernate (Hib.). In Hibernate mode, both the transceiver and the processor are powered down. Only a quartz crystal unit is powered, allowing the processor to wake up and then to switch on the radio stage.

**Figure 8.**
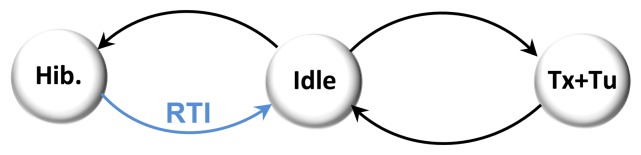
Telemeter state machine.

The system is woken from the Hibernate state every 256 ms by the real time interrupt (RTI) timer to manage pending commands (Idle). For each wake up, the Telemeter broadcasts an RF localization request (Tx) and generates an ultrasonic pulse (Tu) before returning to the Hibernate state. Each transition of the state machine goes through the Idle state. The state machine is described in Figure 8.

#### 4.1.2. Beacon Software

**Figure 9.**
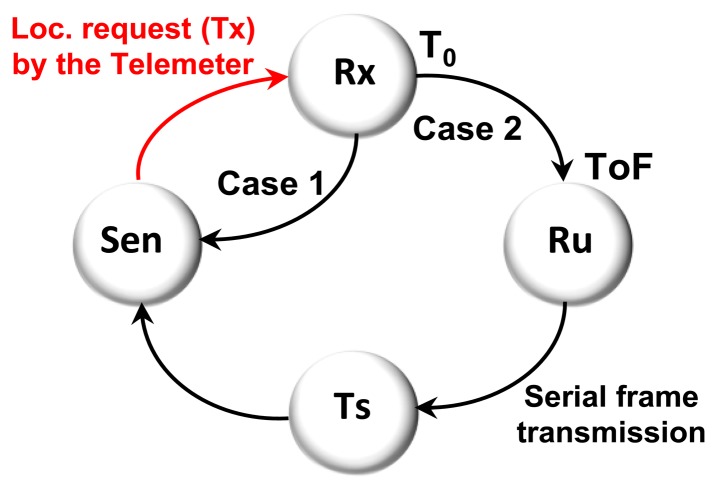
Beacon state machine.

The Beacon node is always in reception mode sensing (Sen) localization requests. As soon as the Beacon node receives (Rx) a localization request (Tx) by the Telemeter, a timer is started (T0). The Beacon waits for an ultrasonic pulse; at this level two cases are possible, as shown in Figure 9:

- Case 1: The ultrasonic pulse is not received and the watchdog expires after 60 ms and the system returns to the Sen state.
- Case 2: The ultrasonic pulse is received (Ru), so a ToF measurement is performed in order to provide the distance between the Telemeter and the Beacon. The Beacon transmits the serial frame (Ts) to the Ethernet board that contains the ToF and returns to the Sen state.

Each transition of the state machine goes through the Idle state.

### 4.2. Frame Format

#### 4.2.1. Radio Frames

**Figure 10.**
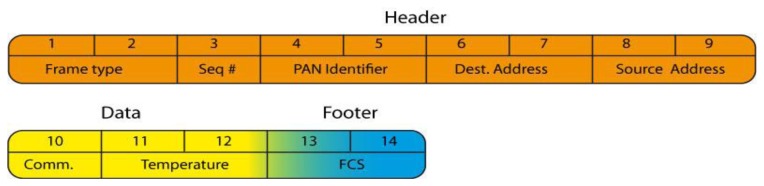
Radio frame format.

The radio frame format uses the 802.15.4 standard header and adds certain fields. Frames are between 12 and 14 bytes long and are composed of three parts as described in Figure 10.

The frame includes:

- The header field: these bytes are specific to the 802.15.4 communication protocol. Details are given in our previous publication [16].
- Data field: byte 10 identifies the command type. For this application only one command is implemented, the Localization Request. Bytes 11 and 12 enable the temperature to be sent from the Telemeter to the Beacon node in order to take the US wave propagation speed compensation into account. This field can be used to develop parameters for other non-implemented commands.
- The footer field: bytes 13 and 14 are generated automatically by the data transmission primitive implemented in the SMAC code base. The FCS enables frame error detection.

#### 4.2.2. Serial Frames

**Figure 11.**
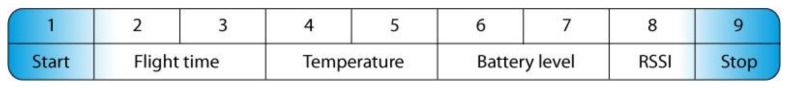
Serial frame format.

After the Beacon has computed the ToF, a serial data frame is sent to the Ethernet board from the MPU. The format of the frames exchanged between the MPU and the Ethernet board is shown in Figure 11. The three Beacons send a frame allowing the position of the Telemeter to be extracted by the trilateration method.

In this process:

- byte 1 is used as a start frame delimiter in order to limit erroneous frames;
- bytes 2 and 3 transmit the US wave flight time;
- bytes 4 and 5 transmit the Beacon temperature information in order to correct the propagation speed of the ultra-waves;
- bytes 6 and 7 transmit the battery level from the integrated battery monitoring system;
- byte 8 gives the link quality indicator, which will enable us to compute the receive signal strength indicator (RSSI);
- byte 9 is used as a stop frame delimiter in order to limit erroneous frames.

### 4.3. Real Time Application

**Figure 12.**
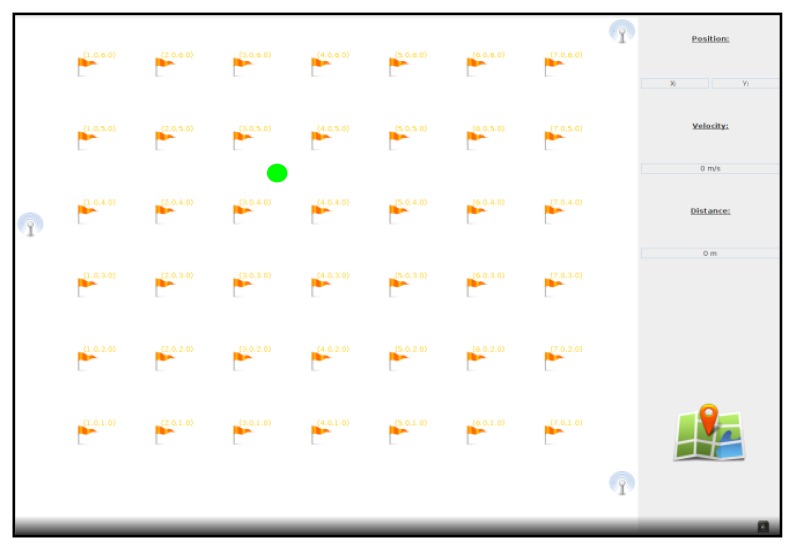
Real time application.

The real-time application developed in JAVA uses the ToF computed by the three Beacons in order to locate the Telemeter in our laboratory room using the trilateration method. To perform our measurements, the dimensions of the laboratory room were recorded using 2D Cartesian coordinates. The coordinates of each Beacon were plotted on a virtual map. Reference points have been added in all the meters in order to compare the real and measured positions. The graphical interface presented in Figure 12 displays the Beacon positions, reference points (flags), the current position of the Telemeter (circle point), and the calculation of speed and covered distance at the instant t when the speed is greater than 0.2 m/s.

### 4.4. Post-Processing Software

**Figure 13.**
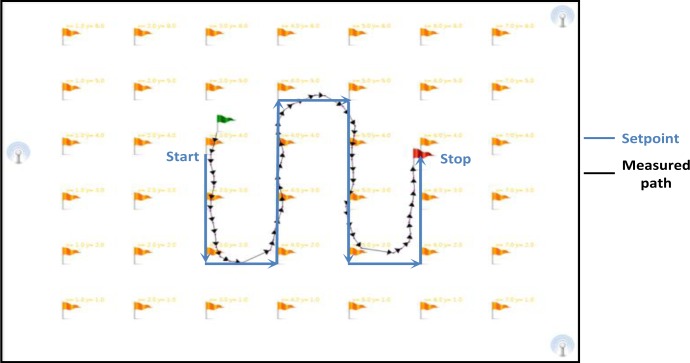
Path filtered by the post-processing software.

The post-processing software developed in JAVA automatically treats the trajectories recorded by the real-time application. The path is filtered and then calculations are made of the total distance covered and the average speed of the path. Figure 13 shows an example of a walking path filtered by the post-processing software. The green flag indicates the start of the path measured, the red flag indicates the stop point, and the blue line is the set point.

## 5. System Characterization

### 5.1. Electrical Consumption

In order to characterize electrically the Telemeter and the Beacon, we measured the current going through a serial 50 Ω resistor before the 9 V to 5 V DC/DC converter.

#### 5.1.1. Telemeter

**Figure 14.**
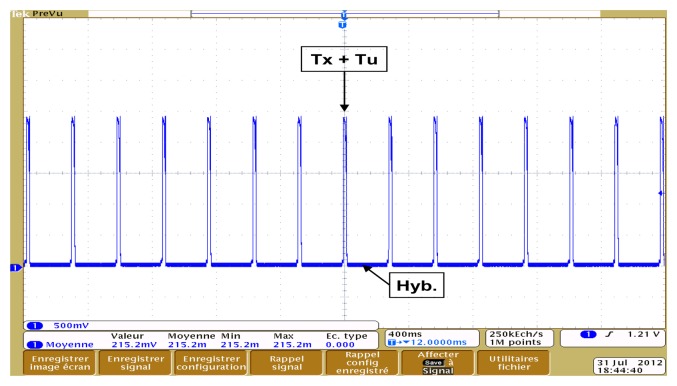
Current consumption of the Telemeter.

We observe in Figure 14 current peaks (48.8 mA) that correspond to sending simultaneously RF and ultrasonic signals (Tx + Tu) every 256 ms. The rest of the time, the Telemeter is in hibernation mode (Hib.) and consumes very little energy (10 μA).

The average consumption of the Telemeter is estimated in Table 1 functioning as the periodic wake-up timer (RTI).

These measures afford a compromise between the periodic localization request and the autonomy of the Telemeter. We have chosen to fix the RTI at 256 ms in order to increase the resolution of the walking path.

#### 5.1.2. Beacon

Energy consumption has not been optimized for the Beacon because it can be powered by the electrical grid. The average consumption is estimated in Table 2 functioning in terms of the periodic localization request from the Telemeter fixed by its RTI.

### 5.2. Localization Characterization

#### 5.2.1. Test Environment

**Figure 15.**
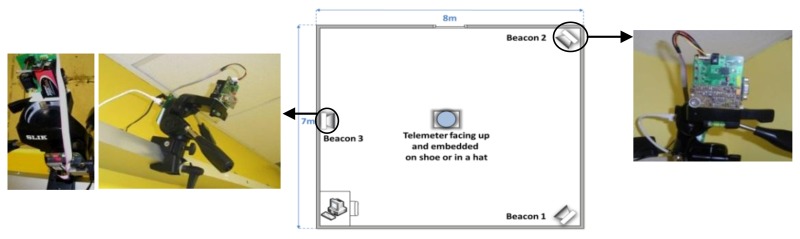
Test room configuration.

The dimensions of the test room were 7 m × 8 m (56 m^2^), the ground and walls are of mixed reflective surfaces (concrete, plaster, *etc.*). Figure 15 shows the room configuration with the positions and orientations of the Beacons and the Telemeter.

**Figure 16.**
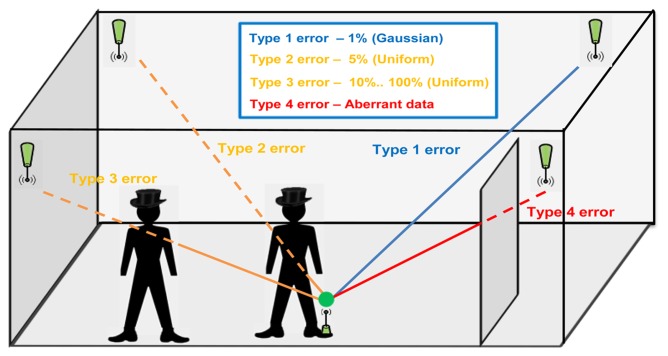
Error sources.

In our experiment, the ultrasonic transmitter of the Telemeter is oriented facing up and the Beacons are inclined to the centre of the laboratory room. In the worst case scenario of this configuration, the misalignment angle between the transmitter and the receiver is around 90°. For a 90° misalignment, the maximum range of the system (Telemeter to Beacon) is 9 m. To remain within the coverage of our system, the monitoring is not undertaken along the walls. The coverage of our system in this configuration is 30 m^2^. An empty room is the ideal scenario to evaluate the performances of a localization system. In a real environment, the major error sources for an ultrasonic system are related to obstacles (furniture, people, *etc.*). A study categorizes these error sources into four types [33], as shown in Figure 16.

- Type 1 is due to temperature drift, hardware delays, or synchronization misalignment, usually following a Gaussian distribution.
- Types 2 and 3 are due to moderate degrees of obstruction of the signal by objects or people around the mobile node. The distribution of obstacles does not follow any particular pattern; these errors can be correctly modeled by a uniform distance-dependent distribution with a maximum value varying from 5% to 100% of the distance.
- A Type 4 error means a complete signal blockage, in which case measured data, if any, will be aberrant.

This analysis is very helpful to design the deployment of the beacons. The most important conclusion of this study [33] is that the system must be designed to ensure that sufficient correct data are available to calculate the location of the mobile node accurately, regardless of the environment. Therefore, the best way of ensuring accurate positioning is to collect redundant data and use a good algorithm, such as the least-median-of-squares algorithm [33], that is able to filter out erroneous measurements.

#### 5.2.2. Static Tests

For these tests, we use the real time application in order to record the coordinates of each measurement. For each reference point, we performed 100 static measurements with the Telemeter alone (not embedded in clothes) and placed on the floor. The average distance error (cm) and the standard deviation (cm) between the real and measured positions were computed for each reference point and the global average distance error (cm) was computed overall for the test area.

**Figure 17.**
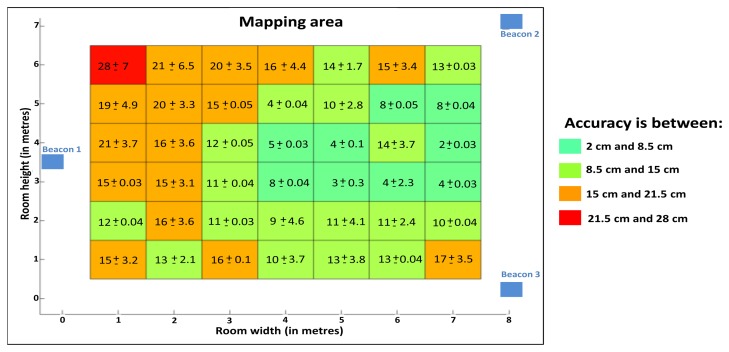
Results of static tests.

We report the results of the average position error (cm) and the standard deviations (cm) on the virtual map. Figure 17 shows the results of static tests for each reference position in the test room.

The average position error varies between 2 cm and 28 cm and the standard deviation varies between 0.03 cm and 7 cm depending on the selected position in the test room. The global average position error is 10.8 cm and the global average standard deviation is 2 cm within the coverage of our system 30 m^2^(5 m × 6 m). We also note that the average position error is larger on the left side of the test room. Indeed, with a single Beacon, the left part of the test room is less well covered and the average position error grows. Thus, positioning a Beacon in each corner of the test room allows the average position error to decrease. The choice of the number of Beacons depends on the application constraints, especially in terms of cost, expected performance and the area to be covered.

#### 5.2.3. Dynamic Tests in Clothes

We assigned 2D Cartesian coordinates on the floor; several tracks are installed on the floor as guidelines. These guidelines are composed of straight lines to facilitate the comparison between the real and estimated paths. These tests are realized with the Telemeter embedded in the clothes of a user. The instruction given to the user was to follow the guidelines at a normal gait speed (∼1 m/s). First, the real time application computes the path using the trilateration method. Then, the post-processing software filters this path and computes the average speed and distance covered. For these tests, we used a chronometer in order to compare the real and estimated measurements of mobility parameters. For each trajectory we measured:

- the average position error (cm) with respect to the guidelines;
- the maximum position error (cm) with respect to the guidelines;
- the average error (%) on the distance covered;
- the average error (%) on the gait speed.

**Figure 18.**
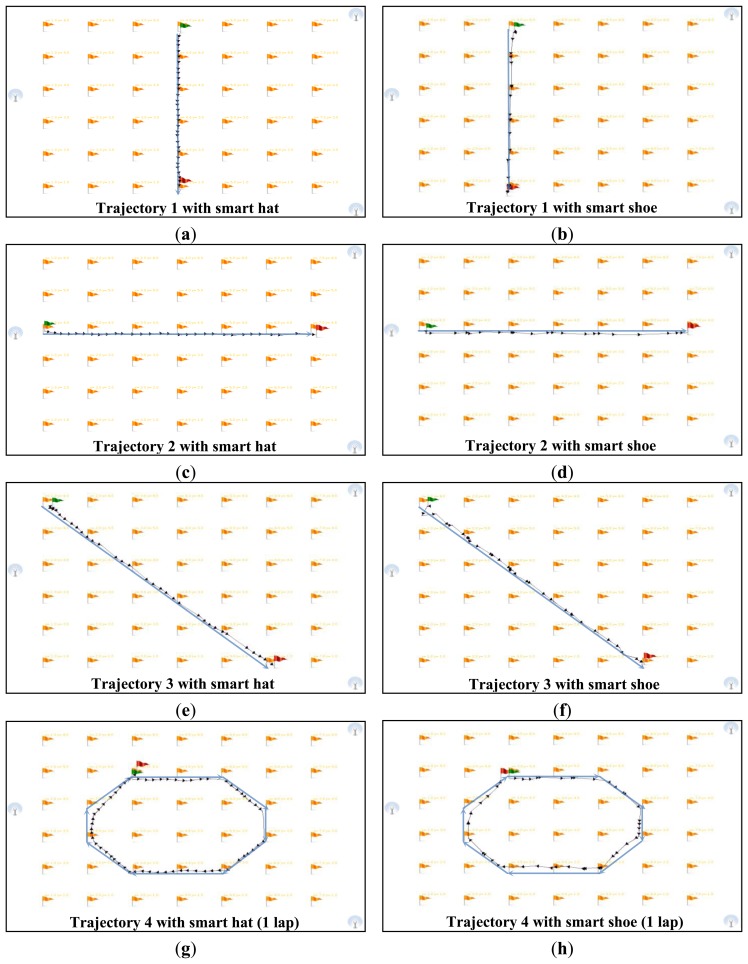
Results of dynamic tests: (**a**) Trajectory 1 with smart hat. (**b**) Trajectory 1 with smart shoe. (**c**) Trajectory 2 with smart hat. (**d**) Trajectory 2 with smart shoe. (**e**) Trajectory 3 with smart hat. (**f**) Trajectory 3 with smart shoe. (**g**) Trajectory 4 with smart hat. (**h**) Trajectory 4 with smart shoe.

We defined several paths to conduct the dynamic tests. The displacements of a person in a living place are generally short, e.g., moving from one room to another, from the sofa to the library, from the table in the dining room to the fridge, *etc.* Thus, paths 1, 2 and 3 are used to measure the system performances on short paths. Path 4 enables measurement of the system performances on the largest path (closed circuit). Figure 18 shows the tested paths using the smart shoe and smart hat after filtering using post-processing software.

Table 3 shows the measurements with the smart hat on all the defined trajectories.

On short paths (1 to 3) the average position error is less than 5 cm and the maximum position error is 15 cm. The average error on the distance and walking speed in the worst case (path 3) is 7.3% and 9% respectively. Indeed, the condition to detect a movement (fixed at 0.2 m/s) implies a significant error on a short path. However, in long-term monitoring, this error implies less drift on the mobility parameters than position errors in a static position.

On path 4, we note that the average position error decreases with the increasing number of laps. Indeed, when the path is long, the position errors offset each other until they give a minimum average position error (4.5%). The maximum position error recorded logically is greater when the number of measurement points is more important. Finally, the average errors on the mobility parameters decrease with an increasing number of laps. Indeed, the condition to detect a movement has less influence on the calculation of these parameters in the case of a long path. Table 4 shows the measurements for the smart shoe on all the defined trajectories.

The comments for the smart hat remain valid for the smart shoe. However, the performances are lower than those for the smart hat. Indeed, in the case of the smart shoe, the human body can be in opposition between the Telemeter placed on the shoe and the Beacons positioned on the ceiling. The path covered by the ultrasound may be longer in some cases which involve some distorted measuring distances. We note in Figure 17 that the number of measurement points defining the different paths is less important than for the smart hat. Indeed, approximately 25% of positions are filtered by the post-processing software against 10% for the smart hat. Resolution is acceptable with an average of three points per second. The accuracy (>90%) seems acceptable in terms of the long-term monitoring of the elderly at home.

## 6. Discussion

The choice of the system position in a garment should be considered in relation to the position of the beacons in the environment. In the case of Beacons positioned on the ceiling, the performance of the localization system is better if the Telemeter is positioned on the head. However, this position is not practical for use in real situations because it requires the user to wear a hat in the home. A Telemeter positioned on the shoe seems more interesting from practical perspective. However, the localization system performances could be lower because the number of obstacles at floor level is larger. Thus, the upper body could be more interesting because there are fewer obstacles. The shoulder is an interesting position in terms of performance and integration in a garment (shirt, sweater, *etc.*). All these issues are currently being studied and a decision will be taken when the final system size is known.

## 7. Future Works

**Figure 19.**
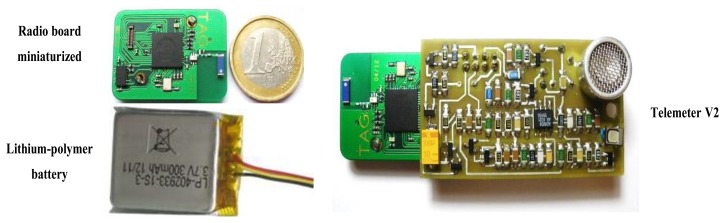
The Telemeter V2.

Currently, we are developing a miniature Telemeter in order to embed it easily into a garment. The radio board has been miniaturized, especially by the replacement of the 9 V battery by a lithium-polymer battery rechargeable (3.7 V, 300 mAh). An accelerometer also has been added in order to measure ADL more precisely. The ultrasonic board is being of miniaturization. The prototype V2 is shown in Figure 19.

In this new configuration, the telemeter can be worn at several locations (e.g., waist, shoulder, shirt pocket, *etc.*). Home tests must be conducted to select the most appropriate location. It is also planned to perform tests in few volunteer homes. To solve the issues related to obstruction of the signal, it seems essential to use more than three Beacons in the main rooms. In order to have a maximum coverage, it seems that a Beacon positioned in each corner of the ceiling and one Beacon positioned in the middle of the ceiling is a good configuration [33]. Thus, Least-median-of-squares algorithm [33], enables to choose the best combination of three Beacons, in order to localize the mobile node correctly.

Finally, in the perspective of system deployment in a multi-user environment, a synchronization protocol combined with an adapted medium access control algorithm would solve interference issues.

## 8. Conclusion

The main objective of this work is to provide a “smart” garment for monitoring the ADLs of the elderly in institutions or at home. This tool is a Telemeter system embedded in clothes measuring certain mobility parameters and allowing precise localization in an indoor environment. Indeed, by combining this garment with a network of anchor points, a local unit allows the displacements of a person to be monitored in an indoor environment and in real time. The Telemeter uses an RF 802.15.4 signal to start the ToF measurement of an ultrasonic emission in order to compute the distances between the device worn by the user and three Beacons fixed in the environment. The localization is computed from these distances using the trilateration method. The coverage of the localization system is 30 m^2^. The characterization of static performances shows:

- good reliability: the standard deviation is 2 cm;
- good accuracy: the average position error is 10.8 cm.

The mobility parameters are computed using a post processing application to filter measurement points inconsistent with the displacement recorded. Tests were performed with the system embedded on a shoe and in a hat. The characterization of mobility performance shows:

- good accuracy on distance covered (92.3% in the worst case);
- good accuracy on gait speed (90.5% in the worst case).

The accuracy in terms of the mobility parameters is acceptable in the context of the monitoring of the elderly at home. Indeed, the goal is to provide certain mobility indicators to health professionals so that they can assess development over the long term. In the case of frail elderly people living at home, these indicators allow earlier intervention in the case of abnormal mobility loss and potentially prevention of further mobility loss.

The Telemeter system shows good energy efficiency: the 802.15.4 low power modes have a capacity of up to one month with standard alkaline batteries (for a measurement every 256 ms). At present, the Telemeter system is in the miniaturization process in order to facilitate easier integration in a garment. Home tests must be conducted to define the location of the devices embedded in the environment and worn by the user. In order to deploy the system in a real environment, algorithms must be implemented to correct the issues from interferences and obstruction of the signal.

## Figures and Tables

**Table 1.** Average current consumption of the Telemeter.

**RTI of the Telemeter (ms)**	**Average current consumption of the Telemeter (mA)**
256	4.3
512	2.9
1,024	2.0

**Table 2.** Average current consumption of the Beacon.

**RTI of the Telemeter (ms)**	**Average current consumption of the Beacon (mA)**
256	19.4
512	24.2
1,024	27.8

**Table 3.** Dynamic tests with the smart hat.

**Smart Hat Trajectories**	**Average Position Error (cm)**	**Maximum Position Error (cm)**	**Average Error on Distance Covered (%)**	**Average Error on Gait Speed (%)**
**Trajectory 1**	0.8	8	5.3	7.6
**Trajectory 2**	1.4	6	6.2	8.1
**Trajectory 3**	4.4	15	7.3	9
**Trajectory 4 (1 lap)**	6.3	26	7.7	9.2
**Trajectory 4 (3 laps)**	5.2	28	4.1	4.9
**Trajectory 4 (5 laps)**	4.9	30	3.9	4.5

**Table 4.** Dynamic tests for the smart shoe.

**Smart Shoe Trajectories**	**Average Position Error (cm)**	**Maximum Position Error (cm)**	**Average Error on Distance Covered (%)**	**Average Error on Gait Speed (%)**
Trajectory 1	3.4	13	6.7	8.2
Trajectory 2	2.8	17	7.5	8.8
Trajectory 3	5.6	26	7.7	9.1
Trajectory 4 (1 lap)	7	38	8	9.5
Trajectory 4 (3 laps)	6.1	34	4.8	5.7
Trajectory 4 (5 laps)	5.4	43	4.5	5.5
